# Evaluation of serum ferritin for prediction of severity and mortality in COVID-19- A cross sectional study

**DOI:** 10.1016/j.amsu.2021.02.009

**Published:** 2021-02-12

**Authors:** Sibtain Ahmed, Zeeshan Ansar Ahmed, Imran Siddiqui, Naveed Haroon Rashid, Maheen Mansoor, Lena Jafri

**Affiliations:** a Department of Pathology and Laboratory Medicine, Aga Khan University. Stadium Road, P.O. Box 3500, Karachi, 74800, Pakistan; b Intensive Care Unit, Department of Medicine, Aga Khan University. Stadium Road, P.O. Box 3500, Karachi, 74800, Pakistan; c Medical College, Aga Khan University. Stadium Road, P.O. Box 3500, Karachi, 74800, Pakistan

**Keywords:** Ferritin, COVID-19, Severe, Mortality, Prognosis

## Abstract

**Background:**

Ferritin even though widely recognized as a representative of total body iron stores, its prognostic utility is linked with COVID-19. This study was aimed at evaluation of the association of ferritin with severity in Coronavirus disease 2019 (COVID-19), hospitalized patients and to test the hypothesis that it is an independent predictor of mortality.

**Material and methods:**

This study was conducted at Chemical Pathology, Department of Pathology and Laboratory Medicine, Aga Khan University (AKU), Karachi. Medical records of all in-patients including both genders, and all age groups with documented COVID-19 from 1st March to 10^th^ August 2020 were reviewed. The subjects were divided into two categories severe and non-severe COVID-19; and survivors and non-survivors. The details were recorded on a pre-structured performa. Between-group differences were tested using the Mann–Whitney's *U*-test. The receiver operating characteristic curve was plotted for ferritin with severity and mortality. A binary logistic regression was used to identify variables independently associated with mortality. The data was analyzed using Statistical Package for the Social Sciences (SPSS)**.**

**Results:**

A total of 336 in patients were reviewed as declared COVID-19 positive during the study duration, and 157 were included in the final analysis including 108 males and 49 females. Statistically significant difference in ferritin was found in the two categories based on severity and mortality. Binary logistic regression showed ferritin to be an independent predictor of all-cause mortality supplemented with an AUC of 0.69 on ROC analysis.

**Conclusions:**

Serum ferritin concentration is a promising predictor of mortality in COVID-19 cases.

## Highlights

• Serum Ferritin was evaluated as a prognostic biomarker in COVID-19
• Statistically significant difference in ferritin was found in the two categories based on severity and mortality.
• Ferritin was found to be an independent predictor of all-cause mortality

## 1 Introduction

The novel coronavirus disease 2019 (COVID-19) that emerged in December 2019 in Wuhan (Hubei, China), has surprisingly occupied the entire globe overwhelmingly, with many countries experiencing the second wave [1]. Even though the rapidly evolving clinical course and presentation continue to amaze the medical fraternity, cases infected with this severe acute respiratory syndrome coronavirus 2 (SARS-CoV-2), often present with severe pneumonia and organ targeted injuries involving the liver, heart, and kidneys [2].

With the surging devastating effects of the pandemic, the focus of scientific efforts was on developing optimal therapeutic regimens to combat the virus. Meanwhile, there was also a dire need for early risk stratification systems and biomarkers to predict disease progression, to identify high-risk patients at an early stage of the infection [[3], [4], [5]]. This can optimize management goals, and overcome the shortage of medical and material resources which was particularly evident amidst this global emergency.

The primary triggering event, associated with severity and mortality has been the inflammatory cytokine storm, characterized by abrupt and excess release of pro-inflammatory cytokines including inflammatory cytokines released by macrophages particularly the interleukins IL-6, IL-10, and tumor necrosis factor (TNF-α) [6]. With this pivotal event of the pathophysiological mechanism in perspective, biochemical analysis of plasma inflammatory markers and positive acute phase reactants including ferritin could be useful for predicting the disease progression [7]. Ferritin occurs as a cytosolic protein in most tissues, although a mitochondrial form also exists and nuclear localization has been proposed [8]. Even though widely recognized as a representative of total body iron stores, its prognostic utility is linked with acute and chronic inflammatory processes and is nonspecifically raised in a variety of such disorders, including chronic kidney disease, rheumatoid arthritis, and autoimmune disorders, etc. [9].

In one study from China with twenty COVID-19 cases, it was found that individuals with severe diseases often present with increased serum ferritin levels, with a statistically significant difference between severe and mild categories [10]. Whereas another study conducted using records from a large multi‐hospital New York City health system demonstrated poor performance of serum ferritin for the prediction of mortality [11].

Owing to the scarcity of literature on the role of ferritin, and the contrasting results demonstrated by various studies on the potential association with severity and mortality in context, this study was aimed at evaluation of the association of the biomarker with severity in a cohort of hospitalized cases with COVID-19 at a tertiary care referral center in Karachi, the metropolis hardest hit by the pandemic in Pakistan.

## 2 Material and Methods

This retrospective observational study was conducted at the section of chemical pathology, department of Pathology and Laboratory Medicine, in collaboration with the section of molecular pathology and intensive care unit, department of Internal Medicine, Aga Khan University (AKU), Karachi, Pakistan. The hospital was the first to launch treatment facilities for COVID-19 in Pakistan. Being accredited by the Joint Commission International (JCI), the hospital serves a population from across the country. The hospital houses the leading infectious disease consultants of the country alongside dedicated areas for COVID-19 screening and treatment with optimal infection control practices. The study was approved by the institutional ethical review committee of the AKU, Karachi (ERC#2020-5168-14099). Our study was registered with Chinese Clinical Trial Registry (Registration No: ChiCTR2100042375). This work has been reported in line with the STROCSS criteria [12].

Medical records of all in-patients including both genders and all age groups with SARS-CoV-2 positive on a reverse-transcriptase polymerase chain reaction (PCR) test from 1st March to 10th August 2020 were reviewed. The PCR specimen was collected on a nasopharyngeal swab using Cobas® SARS‐CoV‐2 Qualitative assay for use on the Cobas® 6800/8800 Systems (Roche Molecular Systems).

The demographic details, length of stay in the hospital, and outcome (survived or expired) at the time of discharge along with the results of ferritin levels were recorded on a pre-structured questionnaire. Of all the COVID‐19‐positive patients, only hospitalized patients aged 18 years and older with a ferritin level available over admission were included in the analysis. Owing to the limitations of retrospective data analysis, ferritin levels obtained within 48 h of admission were acquired. If multiple ferritin levels were obtained, then the one closest to admission was recorded. Serum ferritin was measured by Chemiluminescence immunoassay (CLIA) on the Siemens Advia Centaur immunoassay analyzer using the manufacturer's recommendations. Results are expressed as nanogram of ferritin per microliter of serum (ng/mL). For internal quality control, 2 levels of manufacturer-provided controls (low and high) were run with each batch of analyte while the laboratory is accredited by the College of American Pathologists (CAP), ensuring optimal external quality assurance.

For assessing the role of ferritin in mortality prediction, the study sample was further divided into two groups i.e. survivors and non-survivors. Likewise, two groups were formulated to assess' severity, being defined as having either of the two criteria; requiring intensive care admission or assisted respiration based on invasive ventilation or non-invasive oxygen support.

The data was analyzed using Statistical Package for the Social Sciences (SPSS) version 26 (IBM Corp., Armonk, NY). As the data that was skewed; median values were reported along with interquartile ranges (IQR) for continuous variables. Between-group median differences were tested using the Mann–Whitney's *U*-test for continuous. P < 0.05 was considered statistically significant and P < 0.01 as highly significant. The receiver operating characteristic curve (ROC) was plotted to further appraise the relationship of ferritin with severity and mortality respectively and the area under the curve (AUC) calculated. The cut-off values were determined as the maximum value giving the best balance between sensitivity and specificity. Moreover, a binary logistic regression was also used to evaluate the association with mortality.

## 3 Results

**Fig. 1:**
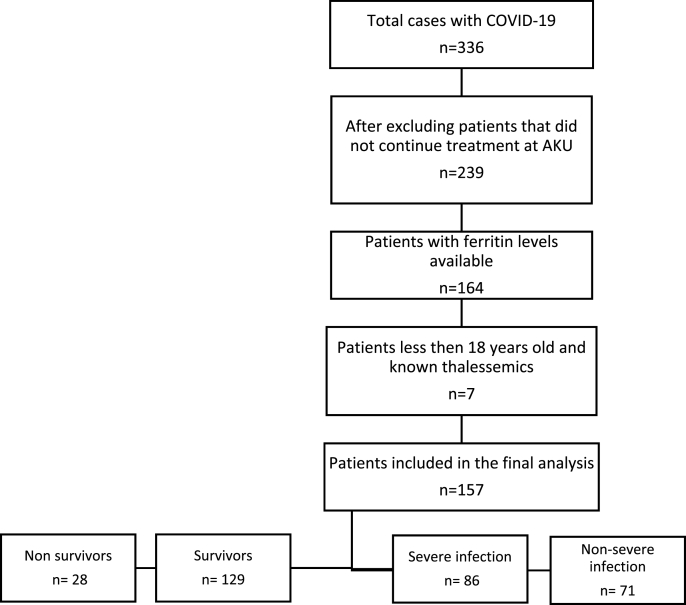
Flow diagram of data scrutiny of cases.

A total of 336 inpatients were reviewed as declared COVID-19 positive during the study duration. Ninety-seven cases i.e. 29% were excluded in the data collection phase as they opted out from their treatment at AKU after a positive COVID-19. The study sample was further scrutinized based on the exclusion criteria and a total of 157 cases were included in the final analysis as shown in Fig. 1. There were a total of 86 (55%) cases in the severe category and out of these 28 (33%) progressed to mortality. Whereas, 71 (45%) of the non-severe category survived.

The demographic details, length of hospital stay, and serum ferritin levels in the severe and non-severe groups and survivor and non-survivor groups respectively are depicted in Table 1 and Table 2. Median ferritin being 828.5(IQR: 428.5–1386.7) and 357.5(IQR: 198.91098) ng/mL was found to be significantly higher in the severe group compared to the non-severe cases group respectively (p value = 0.005) however the association with severity was lost on binary logistic regression analysis (p value = 0.082). Whereas, the ferritin levels with a median value of 1096.4 ng/mL in the non-survivor group, were found to be significantly higher compared to survivors (p-value = 0.02) and was found to be an independent predictor of mortality on binary logistic regression (p-value = 0.02). Furthermore, older age and increased duration of hospital stay were also revealed as variables independently associated both with severity and mortality as shown in Table 1, Table 2.

**Table 1:** Case details and ferritin in Severe Vs Non-Severe group.

	Severe Cases (n = 86) M:F (60:26)	Non-Severe Cases (n = 71) M:F (48:23)	Mann–Whitney's *U*-test *(p-value)*	Binary logistic regression *(p-value)*
**Age in years (Median IQR)**	59.5 (52–70)	54 (39–68)	0.528	**0.01**
**Length of hospital stay in days (Median IQR)**	13 (8–20)	8 (4–12)	**0.001**	**0.002**
**Ferritin in ng/mL (Median IQR)**	828.5 (428.5–1386.7)	357.5 (198.91098)	**0.005**	0.082

P < 0.05 statistically significant and P < 0.01 highly significant.

**Table 2:** Case details and ferritin in Survivor Vs Non-Survivor group.

	Survivor Cases (n = 129) M:F (87:42)	Non-Survivor Cases (n = 28) M:F (21:07)	Mann–Whitney's *U*-test *(p-value)*	Binary logistic regression *(p-value)*
**Age in years (Median IQR)**	56 (44.5–66)	65.5 (57.25–76.25)	**0.008**	**0.0001**
**Length of hospital stay in days (Median IQR)**	9 (6–14)	17 (5–25.75)	**0.022**	**0.012**
**Ferritin in ng/mL (Median IQR)**	548.9 (248.1–1137.9)	1096.4 (609.75–1614.5)	**0.02**	**0.024**

P < 0.05 statistically significant and P < 0.01 highly significant.

**Fig. 2:**
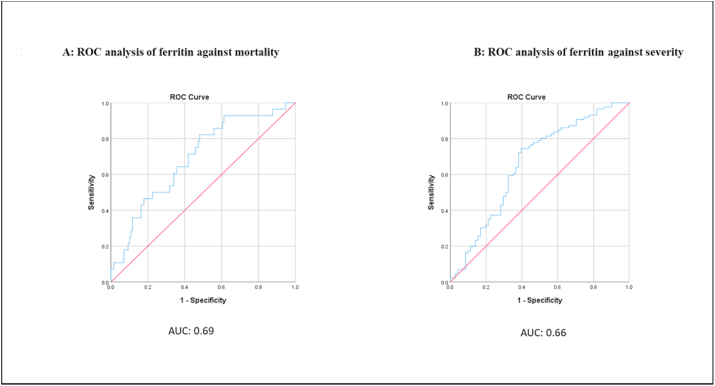
ROC analysis for ferritin against severity and mortality.

**Fig. 3:**
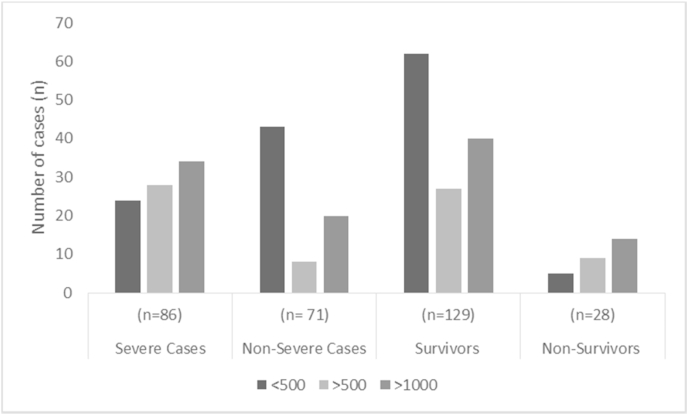
Distribution of cases (n) with hyperferritinemia in the four study groups.

ROC curve analysis was used to compare the performance of ferritin as a predictor of mortality and severity. Ferritin was a slightly better predictor of mortality than severity, with an AUC of 0.69 (95% CI: 0.58–0.79) and 0.66 (95% CI: 0.57–0.74) respectively as illustrated in Fig. 2. The optimal cut-off for prediction of mortality was 574.5 ng/mL with a sensitivity of 82% at the cost of specificity i.e. 51%. Whereas, for the prediction of severity the optimal cut-off identified was 354 ng/mL with a sensitivity of 80% at a compromised specificity i.e. 50%. The study sample was further categorized into three categories based on serum ferritin levels <500, >500, and >1000 ng/mL, and the case distribution according to severity and mortality was assessed as shown in Fig. 3. In the third category of >1000 ng/mL most of the severe cases (n = 39, 40%) and the majority of the expired cases (n = 14, 50%) were placed.

## 4 Discussion

During the global COVID-19 pandemic, the role of laboratory medicine for aid in clinical decision making was mainly focused on prompt risk stratification for smart resource allocation in already exhausted as well as resource-constrained setups in the case of low middle-income countries (LMIC) like Pakistan. Of the various biomarkers evaluated by clinicians based on prior experience of viral pathogens majority were pro-inflammatory cytokines including IL-6, Procalcitonin, lactate dehydrogenase, and C-reactive protein. However, from a laboratorian's perspective, the majority of the small lab set up particularly in LMIC does not have most of these biomarkers readily available on their testing menu. Whereas, ferritin being a commonly used biomarker in hematologic disorders is widely available and it is relatively inexpensive. The logistics, availability, and financial benefits pose ferritin as a potential tool for COVID-19 risk stratification.

This retrospective analysis of 157 COVID‐19 patients admitted to a large tertiary referral center, shows that ferritin levels, either obtained at presentation or near to admission, are a relatively nonspecific test in predicting the evaluated outcomes, namely all‐cause mortality, and severity. This was shown via several analytic techniques, including ROC analysis obtained at optimal cutoff ferritins. There seemed to be some moderate predictive power in ferritin being able to discriminate all‐cause mortality with an AUC of 0.69 and a significant association on binary logistic regression. Likewise, Bennouar S et al. have also reported an AUC of 0.63 for severity in prediction in a cohort of 330 Algerian patients with COVID-19 in the period between the 27th of March and 22nd of April 2020 [13]. Another study by Jonathan et al., based on a retrospective review of 942 adult COVID-19 cases from New York city health system database, have also reported almost similar AUC for mortality and severity in COVID-19, being 0.63 and 0.68 respectively [14].

Pastora J et al. in a systematic review on the utility of ferritin in COVID-19 has revealed that ferritin concentrations of COVID-19 patients were generally within the normal range of less than 400 ng/ml in patients with the non-severe disease [7]. However, hyperferritinemia (ferritin level > 400 μg/L), was observed in patients with a severe disease on admission, precisely between 1.5 and 5.3 times higher in patients. In conjunction, our study also exhibited significantly higher results in the severe category of cases. Pastora J et al. also evaluated studies comparing ferritin levels on admission between COVID-19 patients between survivors and non-survivors and demonstrated that non-survivors showed ferritin levels on admission around 1400 ng/mL, which is between 3 and 4 times higher than that observed in survivors. Likewise, our findings with a median ferritin of 1096.4 (IQR: 609.75–1614.5) ng/mL in the all-cause mortality group was coherent with similar studies reported from China.

Our study reported a male predominance with COVID-19 (n = 108, 69%). Likewise, a retrospective cohort study of 239 hospitalized COVID-19 cases from Lombardy, Italy reported 71% of cases being males and another report from Wuhan China reported 75% [15,16]. Furthermore, the increasing of the patients was an independent predictor of mortality as well as a significant association was noted with severity, with the median age being 65.5 years and 56 years in the non-survivors and severe category. Results reported by Luo Xiaomin et al. in 298 COVID-19 cases from China spanning from 30 January to 20 February 2020 s our findings, where a high proportion of mortality was noted for the age group above 60 years and increasing age was linked with disease advancement [17]. In cohesion with our findings, the global literature has also demonstrated a high association of mortality with older age and mortality in COVID-19 [18,19].

The strengths of this study encompass the large patient population from a COVID hit metropolis, with an optimal number of patients achieving our outcomes of interest and power of the study within a relatively short span of time. Our study certain limitations, mostly owing to its retrospective nature. Less than half of the patients in our original data set had a ferritin value recorded, the missing set if included could potentially affect our findings. This study only evaluated ferritins drawn within 2 days of presentation, we did not explore the impact of serial ferritins over time and how changes in ferritin values could predict outcomes. Moreover, pre‐COVID‐19 ferritin levels were only available for one patient who was a known thalassemia case and was excluded.

## 5 Conclusion

On admission ferritin concentration is a promising predictor of mortality, though, it cannot reliably predict severity. However, owing to statistically significant results obtained and its widespread availability, it is a useful marker of risk scarification in COVID-19 and can be considered in combination with clinical details and other laboratory tests while designing the patient centered treatment plans.

## Annals of medicine and surgery

The following information is required for submission. Please note that failure to respond to these questions/statements will mean your submission will be returned. If you have nothing to declare in any of these categories then this should be stated.

## Please state any sources of funding for your research

None.

## Ethical approval

The study was granted exemption by the institutional ethical review committee of the AKU, Karachi (ERC#2020-5168-14099)

## Consent

N/A.

## Author contribution

Sibtain Ahmed performed the literature search, data analysis and write-up of the work in the first draft. Zeeshan Ansar Ahmed, Imran Siddiqui and Naveed Haroon Rashid were involved in the laboratory workup, patient selection and critical revision of the article for the intellectual content. Maheen Mansoor performed data collection, clean up and helped with tables. Lena Jafri conceived the idea, coordinated the writing of the paper and reviewed the final draft. All the authors have accepted responsibility for the entire content of this submitted manuscript and approved submission.

## Registration of research studies

1. Name of the registry: Chinese Clinical Trial Registry
2. Unique Identifying number or registration ID: ChiCTR2100042375;
3. Hyperlink to your specific registration (must be publicly accessible and will be checked): http://www.chictr.org.cn/showprojen.aspx?proj=120493

## Guarantor

Dr Lena Jafri.

Assistant Professor & Section Head Clinical Chemistry.

Department of Pathology and Laboratory Medicine.

The Aga Khan University, Pakistan.

Phone: 92-213-4861927.

Email: lena.jafri@aku.edu.

## Funding

None.

## Declaration of competing interest

None.
